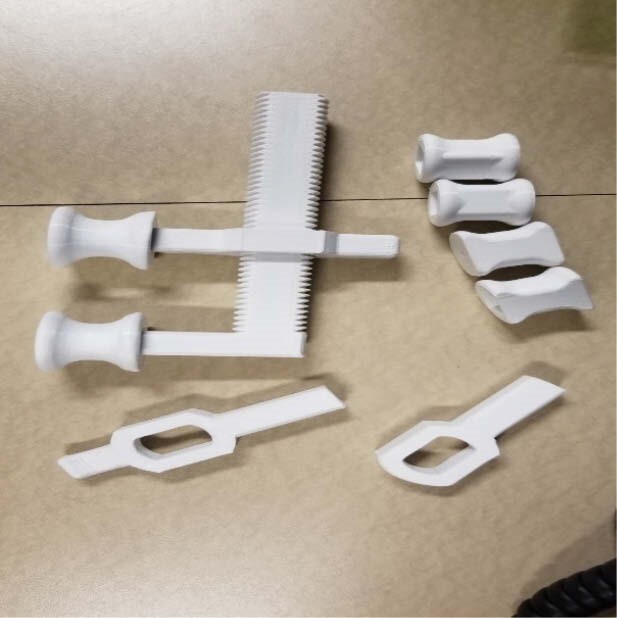# 755 3D Printed Mouth Stretcher

**DOI:** 10.1093/jbcr/irad045.230

**Published:** 2023-05-15

**Authors:** Jana Almendinger, Jennifer Rosenstiel

**Affiliations:** Hennepin Healthcare, Cedar, Minnesota; Hennepin Healthcare, Saint Paul, Minnesota; Hennepin Healthcare, Cedar, Minnesota; Hennepin Healthcare, Saint Paul, Minnesota

## Abstract

**Introduction:**

Microstomia can impede eating, speech, dental care, and surgical procedures (intubation). MPAs (microstomia preventative appliance/apparatus) are often used to manage burns around the mouth where there can be tightness in the oral commissures (microstomia). MPAs, however, are becoming more difficult to attain. Burn center therapy departments have been trying to find, or develop, a comparable replacement. Having been able to procure a 3D printer, our department is now able to print this new design. It has been trialed with a patient who reports the device is comfortable to wear and easy to use.

**Methods:**

In developing a device to incrementally spread the oral commissures, different “garage tools” (C-clamp, caliper, wrench, etc) were trialed. From these ideas, this ratcheting slide version was determined to be the most durable and simple to use. The “clamp” ends were replaced with interchangeable “spools”. It is printed with a plastic filament called PLA (polylactic acid) and took between 100 minutes to 200 minutes to print all pieces of the mouth stretcher depended on the size of the device. The total cost to print ranges from $1.00 to $2.00 per device depending on the size. The main support, the slider, and the spools each need their own STL file, but were designed synchronously so that the parts all fit together properly. This mouth stretcher can be resized via different sized spools or made on a smaller scale for a pediatric version. It can also be used as a horizontal mouth stretcher (instead of using layers of tongue depressors).

**Results:**

Our adult patient was able to improve his oral commissure measurements and he reports the device was comfortable and he was even able to sleep with it. The patient was able to increase the horizontal opening of his mouth 1 cm and vertical opening of his mouth 1.25 cm with inconsistent use over 9 months as he needed to take breaks due to another eye surgery during this time.

**Conclusions:**

Stretching the oral commissures can be done cost-effectively (once a 3D printer is available) and easily used with minimal instruction for the patient. It could be trialed with varying sizes and shapes of “spools” for improving patient comfort and to minimize any areas of tissue breakdown (pressure sores) in the mouth. Past use of microsomia splints have been very beneficial, increasing mouth ROM which can diminish likelihood of surgical reconstruction and decrease the possibility of eversion of lower lip.

**Applicability of Research to Practice:**

It may be difficult to provide for patients due to the expense of procuring a 3D printer and requires knowledge of 3D printing. However, as 3D printing becomes more commonplace in schools, hospitals, and homes, there will be increasing opportunity for further creativity, innovation, and use in the burn world.